# Damage to the Endotracheal Tube Caused by Incessant Biting by an Unconscious Patient After Stroke: A Case Report

**DOI:** 10.7759/cureus.65599

**Published:** 2024-07-28

**Authors:** Mohammed A Ageel

**Affiliations:** 1 Department of Surgery, Jazan University, Faculty of Medicine, Jazan, SAU

**Keywords:** tube leakage, biting, unconsciousness, stroke, airway exchange, endotracheal tube

## Abstract

Endotracheal intubation, a procedure performed using an endotracheal tube (ETT), has been identified as one of the most viable and common methods of managing the airway and artificially supporting respiration. Patient consciousness is an essential factor that is directly linked to airway safety, and an acute drop in the level of consciousness might threaten the airway. A Glasgow Coma Scale score of less than 9/15 is an indication of the need to protect the airway by conducting the commonly known procedure of endotracheal intubation. In the current case report, we found an unusual cause of leakage in the ventilator tube that affected the tube integrity: the involuntary tube biting of a patient admitted to the intensive care unit due to low consciousness provoked by an ischemic stroke. This constitutes an interesting phenomenon that must be investigated further. Aside from deciphering the underlying subconscious event, mitigatory mechanisms should be deployed along with ETT to prevent the ventilator circuit from failing.

## Introduction

Maintaining a patient’s airway and promoting breathing are priorities in breathing-related emergencies. A thorough examination is crucial to safe airway care, particularly in guaranteeing patency. Recognizing a problematic airway is the first step in this process. When a patient is unable to breathe on their own, initial oxygenation and ventilation should be administered using a bag-valve mask, with or without a Mayo cannula, followed by orotracheal intubation performed by a skilled emergency clinician. In a “cannot intubate, cannot ventilate” circumstance, surgical solutions, such as cricothyroidotomy or tracheostomy, should be considered. In addition, long-term artificial ventilation patients will need a tracheostomy. Mechanical ventilators facilitate the movement of air into and out of a patient's lungs while reducing the physical effort required [1,2].

An acute ischemic stroke occurs when a clot blocks a brain artery. Aspiration pneumonia is a common cause of respiratory failure following a stroke and is linked to decreased consciousness, swallowing difficulty, and dysphagia [3,4]. A high incidence of respiratory problems in stroke patients requires appropriate ventilator control [5]. Neurological conditions, such as a Glasgow Coma Score (GCS) of 9, increased intracranial pressure, generalized (tonic-clonic) seizures, an infarct affecting more than two-thirds of the middle cerebral artery territory, and a midline shift observed on imaging, can sometimes necessitate intubation [6-9]. As with any critically ill patient, failure in oxygenation or ventilation is an indication of the need for endotracheal intubation following an acute ischemic stroke [10].

Endotracheal intubation is considered a form of definitive airway management, and it can be performed either through the mouth or nose, that is orotracheal or nasotracheal intubation. Endotracheal intubation, a procedure performed using an endotracheal tube (ETT), has been identified as one of the most viable and common methods of managing the airway and artificially supporting respiration [2].

Notably, ETT accidents are often accompanied by twisting, obstruction, or damage to the tube’s integrity [11]. An Australian study reviewed 189 incidents related to ETT issues, finding that 42% were due to endobronchial intubation, obstruction, and esophageal intubation (each accounting for 18%), disconnections and leaks (7% each), other misplacements (4%), inappropriate tube selection (3%), cuff herniation (1%), and failures to deflate the cuff and foreign objects in the tube (0.5% each) [12]. Data from the National Center for Health Statistics indicated that 1,434,349 individuals died from acute respiratory failure (ARF) between 2014 and 2018, with mechanical ventilation being a primary intervention to maintain adequate breathing [13].

While the reasons for such failures are innumerable, an aspect that is considerably less researched is the case of individuals who suffered from stroke followed by lack of consciousness, which has led them to chew the ETT to the point of sabotaging the ventilator circuit. Establishing a direct correlation between the patient’s unconsciousness and the biting phenomenon requires further study. The adoption of ETT for patients who have lost consciousness due to stroke requires adequate safety measures to compensate for the biting tendencies in such patients. In this case report, we described the loss of conventional ETT integrity due to biting by a stroke patient with a decreased level of consciousness, followed by tracheostomy intubation in an intensive care unit (ICU).

## Case presentation

A 66-year-old male, known to have type II diabetes and hypertension, presented to the emergency department (ED) approximately 20 hours after he was first observed to have a drop in consciousness. At the time of presentation, his initial GCS score was evaluated at 7/15. Clinical assessment revealed dehydration and hypotension. Hemodynamically, the patient's heart rate was within normal limits, and both blood pressure and mean arterial pressure (MAP) were stable. Due to the critical drop in GCS, airway protection was promptly managed through orotracheal intubation using a 7.0-mm cuffed endotracheal tube (ETT) (Mallinckrodt/Covidien, Mansfield, Massachusetts). Following successful intubation, the patient was connected to mechanical ventilation to support respiratory function.

A computed tomography (CT) scan revealed an acute ischemic infarction involving the basal ganglia bilaterally. A carotid duplex ultrasound study revealed 50% calcified plaque and 70% calcified plaque in the left and right common carotid arteries, respectively. Otherwise, there is no other significant organ involvement.

The patient was admitted to the ICU under sedation and mechanically ventilated. A plan for daily drug interruption and neurological evaluation was established, and a CT scan was scheduled to be repeated within 48 hours, which was done and showed radiological changes compared with the initial CT study. The use of sedative drugs was discontinued, and the patient’s condition remained static, with no improvement in his level of consciousness. Regular checking for cuff leaks was conducted, and no leaks were found based on the ventilator volume or auscultation.

The patient remained without sedation on mechanical ventilation (volume control, synchronized intermittent mandatory ventilation (SIMV), fraction of inspired oxygen (FiO2): 35%, respiratory rate (RR): 16 breaths per minute, tidal volume (Vt): 440 ml, positive end-expiratory pressure (PEEP) 5 cm H2O). The patient manifested a continuous mouth movement (biting the oropharyngeal airway, making it protrude, and chewing), which we interpreted as intolerance to the device (oropharyngeal airway). There were concerns about lip, tongue, and soft tissue injuries, so we decided to remove the device.

On the seventh day after the ICU admission, we noticed a leak in the ventilator circuit. The ETT site was checked and found to be well positioned. The endotracheal cuff pressure was checked several times, and during each check, the balloon pressure was within the target. The chest x-ray showed a well-positioned tube depth and no other abnormal features. Finally, a direct check using the laryngoscope showed no suspicious elements.

The leak progressively increased, so we decided to change the ETT using the same tube size and laryngoscope Macintosh blade size 4. The tube replacement was eventless. The ETT balloon inflated, and the balloon pressure was checked and maintained within the target. The chest x-ray showed a well-sited ETT, and the ventilator leaking alarm disappeared.

**Figure 1:**
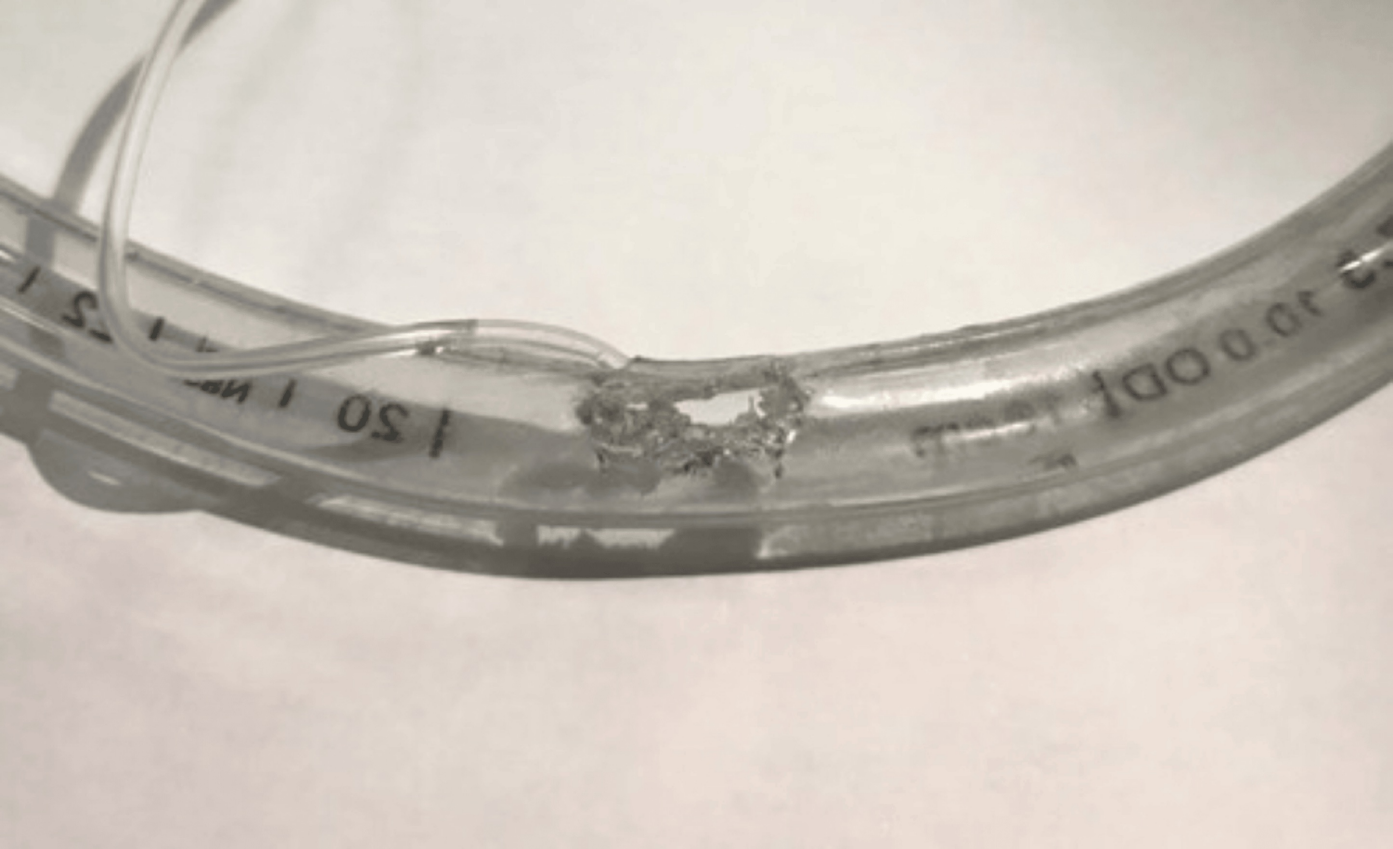
The damaged endotracheal tube bitten by the patient.

The old tube was checked, and we found a hole affecting the tube’s integrity. This hole was irregular and situated deeply, estimated to be at the level of the molars (Figure 1). As there was no improvement in the patient’s consciousness and mouth movement, we decided to opt for a percutaneous tracheostomy.

## Discussion

Endotracheal intubation seems inevitable in emergencies, as it is one of the procedures used to maintain the airway and preserve patency [14]. The concerned individual experienced an acute ischemic stroke at home and lost consciousness pre-hospital admission. Although unconscious, the patient was reasonably stable after the apparatus of conventional ETT was set up. However, he exhibited repeated biting and chewing-like movements on the ETT, generating a hole that affected the tube integrity and, hence, the ventilation circuit.

Patients in a comatose state have often been observed to exhibit certain involuntary physical actions, such as biting, tongue movements, and muscle contractions in various parts of the body [15,16]. Cerebrovascular events are common in the ICU, and either the main vascular injury or the secondary harm that frequently follows owing to edema, mass effect, and disturbance of the brain circuits may cause a wide variety of abnormal movements [17,18]. Various abnormal movements, such as flexor or extensor posturing brought on by severe brain injury and subsequent cerebral edema, may be observed in the comatose state and may represent motor paroxysms in the context of cerebral herniation [15,16]. The abnormal movement, on the other hand, can be a direct result of the underlying primary damage. Additionally, research has linked biting tendencies to the masticatory function, which remains unaffected by the stroke, causing some patients to bite unconsciously [19]. Overall, several physiological and neurological factors have been proposed as potential triggers for such unexpected behaviors.

The patient under question had a failing ventilator circuit on the seventh day of admittance with no possible signs to track the reason. However, the ETT replacement allowed us to discover that the tube had been severely damaged at the junction of the patient’s jaw, which led to the leak of air pressure in the circuit. Transitioning to a tracheostomy was considered a precautionary measure. Several similar cases of conventional ETT biting have been reported by experienced medical teams. However, none of the reported cases involved unconscious patients post-stroke in the ICU. Kaur et al. (2021) reported a similar case for a 34-year-old female patient who was referred after a postoperative radical nephrectomy. Kaur et al. (2021) suggested that bite blocks are most suitable for patients who have slight consciousness or lack strong anesthetics [20]. As per the findings of Reuland (2021), a patient previously experiencing spinal myoclonus suffered from cardiac arrest as the leak in the ventilation circuit overly burdened the patient’s respiratory system; a bite blocker was placed to prevent further ETT obstruction, and a tracheostomy was performed thereafter [21]. In another case, Yoon et al. (2017) reported that a reinforced ETT was severed by a tube bite while the patient was in a prone position during an ICU stay, which required the medical team to surgically remove the dislodged tube from the patient’s right bronchus. According to Yoon et al. (2017), the risks associated with biting should not be taken lightly [22]. Overall, unconscious patients post-stroke require close observation before coming up with a customized solution that best suits them.

## Conclusions

Considering the complications that took place in the case, the ETT deployment should be accompanied by specific safety mechanisms. Apart from open tracheostomy as a feasible alternative, clinicians can consider using oral bite blocks to compensate for the biting tendencies of the patient. Moreover, adopting a reinforced ETT has shown better results in many cases. Overall, adopting ETT as a reliable airway facilitation medium raises many questions that demand better alternatives or medium modifications.
